# Hemin-Modified Multi-Walled Carbon Nanotube-Incorporated PVDF Membranes: Computational and Experimental Studies on Oil–Water Emulsion Separations

**DOI:** 10.3390/molecules28010391

**Published:** 2023-01-02

**Authors:** Ismail Abdulazeez, Billel Salhi, Asma M. Elsharif, Muhammad S. Ahmad, Nadeem Baig, Mahmoud M. Abdelnaby

**Affiliations:** 1 Interdisciplinary Research Center for Membranes and Water Security, King Fahd University of Petroleum & Minerals, Dhahran 31261, Saudi Arabia; 2 Department of Chemistry, College of Science, Imam Abdulrahman Bin Faisal University, Dammam 31441, Saudi Arabia; 3 Chemistry Department, King Fahd University of Petroleum & Minerals, Dhahran 31261, Saudi Arabia; 4 Interdisciplinary Research Center for Hydrogen and Energy Storage (IRC-HES), King Fahd University of Petroleum & Minerals, Dhahran 31261, Saudi Arabia

**Keywords:** oil/water emulsion, antifouling, membrane, PVDF, MWCNT, MD simulation

## Abstract

The separation of oil/water emulsions has attracted considerable attention for decades due to the negative environmental impacts brought by wastewater. Among the various membranes investigated for separation, polyvinylidene fluoride (PVDF) membranes have shown significant advantages of ease of fabrication, high selectivity, and fair pore distribution. However, PVDF membranes are hydrophobic and suffer from severe fouling resulting in substantial flux decline. Meanwhile, the incorporation of wettable substrates during fabrication has significantly impacted the membrane performance by lowering the fouling propensity. Herein, we report the fabrication of an iron-containing porphyrin (hemin)-modified multi-walled carbon nanotube incorporated PVDF membrane (HA-MWCNT) to enhance fouling resistance and the effective separation of oil-in-water emulsions. The fabricated membrane was thoroughly investigated using the FTIR, SEM, EDX, AFM, and contact angle (CA) analysis. The HA-MWCNT membrane exhibited a water CA of 62° ± 0.5 and excellent pure water permeance of 300.5 L/m^2^h at 3.0 bar (400% increment), in contrast to the pristine PVDF, which recorded a CA of 82° ± 0.8 and water permeance of 59.9 L/m^2^h. The hydrophilic HA-MWCNT membrane further showed an excellent oil rejection of >99% in the transmembrane pressure range of 0.5–2.5 bar and a superb flux recovery ratio (FRR) of 82%. Meanwhile, the classical molecular dynamics (MD) simulations revealed that the HA-MWCNT membrane had greater solvent-accessible pores, which enhanced water permeance while blocking the hydrocarbons. The incorporation of the hemin-modified MWCNT is thus an excellent strategy and could be adopted in the design of advanced membranes for oil/water separation.

## 1. Introduction

Water contamination due to organic and oily components has become a critical environmental problem and significantly impacts human and aquatic life [1,2]. Oil can pollute water from several sources, including the frequent oil spills that happen during the transportation of oils, and history has shown some severe oil spill incidents that occurred periodically. Oil and gas extraction industries are the major contributors to oily wastewater. According to the estimation, almost 3 to 10 barrels of produced water are produced with each oil barrel and disposing of it without proper treatment can impact water quality significantly. Other industries produce oily wastewater, including textile, food, mining, and metal processing. Oil water pollution has become a global concern due to its critical implications on the ecological environment [3]. Due to the subject’s significance, oil/water separation has become a hot area of research. 

Oil in water can be present in different droplet sizes, and according to the type or the size of the oil droplet, a membrane design is required. If the diameter exceeds 150 μm, it is called free oil and water; if the droplet size diameter is 20 μm ≤ d ≤ 150 μm, it is called dispersion; and if the diameter is reduced from 20 μm, the term emulsions are used [4,5]. Several methods have been adopted to treat or recover oil, including air flotation, coagulation, skimmers, flocculation, hydro cyclones, and gravity-based separations [6,7]. Various adsorbents have also been developed to remove oily contaminants from the water [8]. The adsorbent becomes saturated after some time and needs to be regenerated. The regeneration may involve chemical or physical steps to make this adsorbent reusable. The overall process becomes tedious if the adsorbent is exposed to highly contaminated water, which demands the fast regeneration or disposal of the materials, which makes the process unfavorable to deal with the large quantity of wastewater [9]. Regeneration of the adsorbent also adds cost to the treatment process [10]. Furthermore, the treatment of oily wastewater has become critical by conventional methods when the droplet size decreases to 20 μm as the oil is emulsified in water [11]. Membrane-based separation of oily wastewater is receiving significant attention due to several desired advantages such as the energy-efficient, low maintenance cost, dealing with large quantities of water, and no addition of immediate secondary contaminants [11].

Due to their controlled pore size, low cost, and facile processing, polymeric membranes have shown great potential for oily wastewater treatment. Therefore, polymeric membranes such as polysulfone [12], polyethersulfone [13], polyacrylonitrile [14], polypropylene [15], and poly(vinylidene fluoride) [16] are being evaluated for oil/water separation. The PVDF membranes are extensively explored for oil–water separation due to their thermal, chemical, and mechanical stability [17]. PVDF is semicrystalline and consists of the repeating unit of –(CH_2_CF_2_)*_n_*–. PVDF can be easily processed into flat sheets, hollow fibers, and tabular and flat sheet membranes. Most of the water is produced by industries; it is oil-in-water emulsions. PVDF is hydrophobic and usually suffers from severe fouling during the separation process, resulting in substantial flux decline. To improve the performance and antifouling characteristics of the polymeric membranes, a range of advanced materials have been used, including graphene [18], graphene oxide [19], g-C_3_N_4_ [20], MXenes [21,22], TiO_2_ [23], ZIF-67 [24], and halloysite nanotube [25]. Carbon nanotubes also display great potential to improve the performance of the polymeric membranes for oil/water separations [26]. Recently, more focus has been observed on utilizing functionalized carbon nanotubes to tune the characteristics of the membranes [27]. 

In the continuation of this efforts, we introduced an iron-containing porphyrin called hemin-modified multi-walled carbon nanotube (HA-MWCNT) to improve the characteristics of the PVDF membranes. The HA-MWCNT was thoroughly investigated theoretically and then experimentally to prove the concept. The HA-MWCNT and the PVDF membranes were investigated by the FTIR, SEM, cross-sections, EDX, elemental mapping, AFM, XRD, and contact angle analysis. The membranes were applied for the separation of the surfactant stabilized oil-in-water emulsions. The modified and the pristine PVDF membranes were evaluated at different pressures to observe the change in flux and rejection while separating the oil-in-water emulsions. The HA-MWCNT membranes maintained a high performance at all evaluated pressures without significantly compromising the rejections. Through computational studies, it was found that the modified MWCNT provided a greater accessible area for the solvent or the continuous phase and thus provided an opportunity to enhance the flux of the membranes compared to the pristine membranes. 

## 2. Results and Discussion

### 2.1. MD Analysis

**Figure 1:**
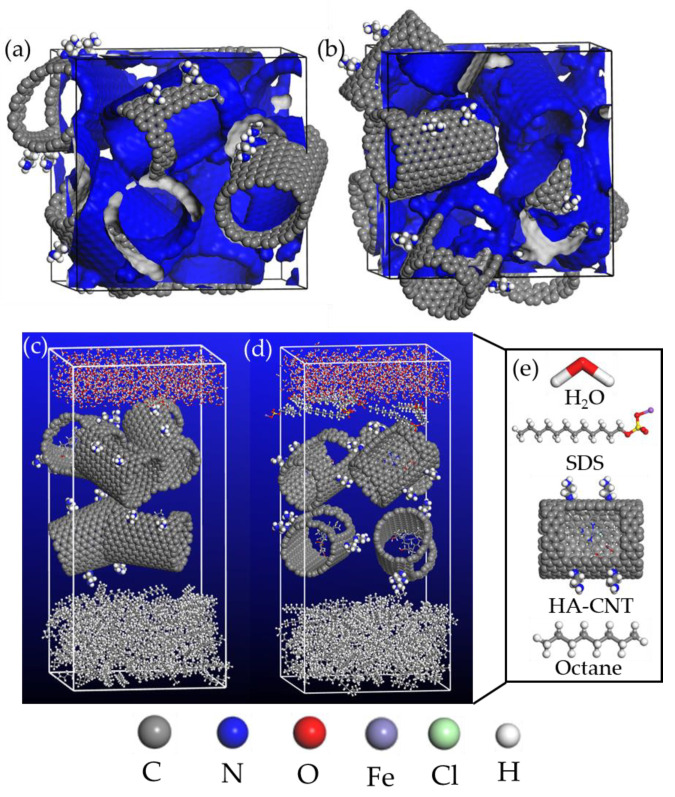
The energy-minimized amorphous cells consisting of 7 units of (**a**) the NH_2_-CNT, and (**b**) the HA-MWCNT active layers. The starting geometry of the (**c**) membrane-oil–water and the (**d**) emulsified membrane-oil–water systems. The respective components of water, surfactant, membrane active layer, and the oil model are shown in (**e**).

Figure 1a,b presents the energy-minimized amorphous cells comprising of seven units of the membrane active layer. The isosurfaces depicted in blue and grey color represents the free accessible volume within the membrane materials at a probe radius of 0.84 Å. Connolly surface area of 20,000 and 25,000 Å^2^ were estimated for the NH_2_-CNT (Figure 1a) and the HA-MWCNT (Figure 1b), respectively. This represents a significant enhancement on the solvent accessibility of HA-MWCNT. Meanwhile, the fractional free volume (FFV) estimated by the Bondi equation [28] resulted in values of 0.296 and 0.305 on the NH_2_-CNT and the HA-MWCNT systems, respectively, in consonant with the Connolly surface area. 

**Figure 2:**
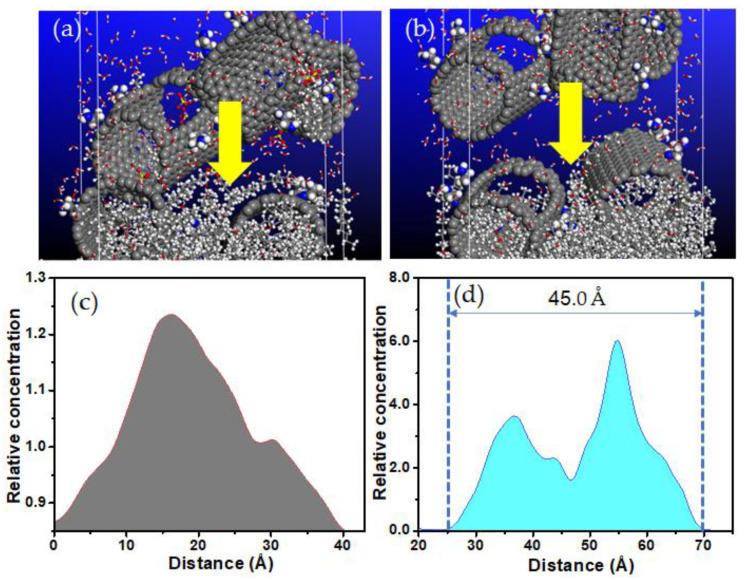
The final frame of the dynamic simulations of the (**a**) membrane-oil–water, and (**b**) the emulsified membrane-oil–water systems. The relative concentration profiles of the membrane-oil–water, and the emulsified membrane-oil–water systems are presented in (**c**,**d**), respectively.

The constructed amorphous cells for the estimation of the oil–water interface stability is presented in Figure 1c,d. The corresponding final frames of the dynamic simulations and the relative concentration profiles are shown in Figure 2. Hydrogen bond interactions between the water molecules and the surface-functionalized amine groups resulted in the aggregation of the NH_2_-CNT at the oil interface which acts as a barrier to oil permeation (Figure 2a). In the emulsified system, however (Figure 2b), a compact layer of the HA-MWCNT was formed which further increased the interfacial thickness. Using the 90–90% criterion [29], interfacial thickness of 40.0 and 45.0 Å were estimated for the membrane-oil–water and the emulsified membrane-oil–water systems (Figure 2c,d), whereas the corresponding interfacial formation energies of −856.2 and −1542.6 kcal/mol were calculated, respectively. These results suggest that the HA-MWCNT membrane material exhibits a larger solvent accessible area and greater FFV, and thus has the potential for the demulsification of oil–water systems.

**Figure 3:**
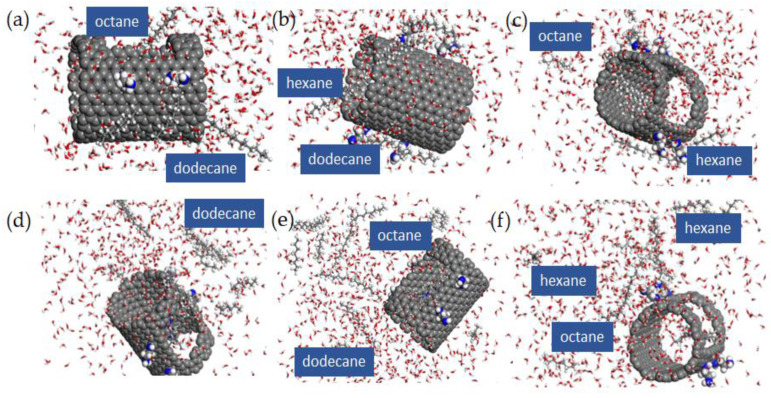
Screenshots of the dynamic simulations of hydrocarbon molecules on NH_2_-CNT at (**a**) 20 ps, (**b**) 100 ps, (**c**) 200 ps. Similar screenshots on the HA-MWCNT at 20, 100, and 200 ps are shown in (**d**), (**e**), and (**f**), respectively.

Furthermore, the interaction of the NH_2_-CNT and HA-MWCNT with various hydrocarbon molecules during the dynamic simulations was revealed by taking screenshots at various time intervals (Figure 3). The achieved data revealed that almost all of the hydrocarbons studied (hexane, octane, dodecane) tend to approach the CNT via the hydrophobic surfaces, while a number of the water molecules interacts via the amine nitrogens. While hexane and octane were able to diffuse through the hollow openings on the NH_2_-CNT (Figure 3a–c), these pathways were inaccessible on the HA-MWCNT due to the gating characteristics of the hemin chloride moieties (Figure 3d–f) leading to the passage of water molecules. These results thus reveal the enhancement in the wettability of the HA-MWCNT due to the intercalation of hemin chloride, and the potential of such material in the separation of oil/water emulsions.

### 2.2. Membrane Characterizations

**Figure 4:**
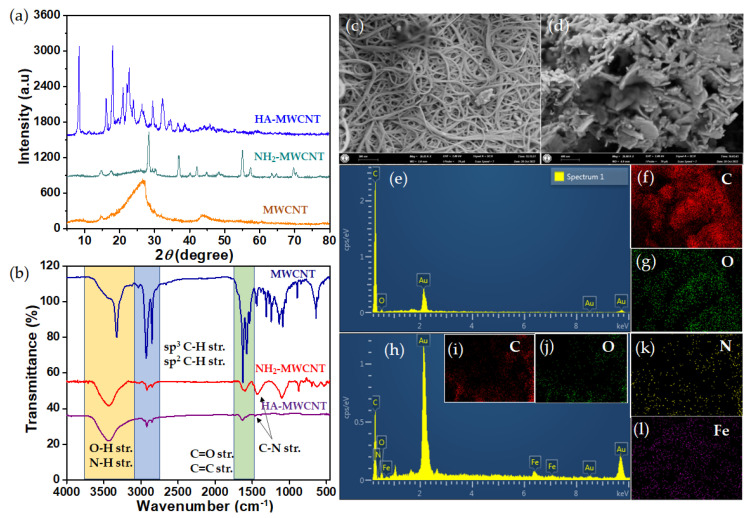
(**a**) X-ray diffraction pattern, and (**b**) FTIR spectra of MWCNT, amine-functionalized MWCNT, and HA-MWCNT. FESEM micrographs of MWCNT and HA-MWCNT are shown in (**c**) and (**d**), while the corresponding EDX and elemental mapping are presented in (**e**–**g**) and (**h**–**l**) for MWCNT and HA-MWCNT, respectively.

The X-ray diffraction (XRD) patterns of pristine MWCNT, the amine-functionalized MWCNT (NH_2_-MWCNT), and HA-MWCNT are depicted in Figure 4a. Accordingly, the same peak at 2*θ* 26.5° corresponding to (002) reflection of graphite provides proof of the presence of carbonized functionality on the pristine and functionalized materials. In the case of NH_2_-MWCNT, we can observe numerous XRD diffraction peaks demonstrating the grafting of the amine on the MWCNT surface. Additional peaks at 14.5°, 19.6° and 37.5° reflecting the (001), (110), and (002) lattice planes, respectively appeared on the NH_2_-MWCNT and the HA-MWCNT affirming the grafting of the amine functionality [30]. Furthermore, the Fe reflections at 7.5°, 21.0°, 30.0°, 32.5°, and 34.0° corresponding to the (112), (113), (220), (104), and (110) lattice planes, respectively [31], revealed the hemin adsorption on the HA-MWCNT.

The FT-IR spectra further revealed the surface groups on the MWCNT, NH_2_-MWCNT, and HA-MWCNT and presented in Figure 4b. The –OH and C=O stretching of carboxylic groups (COOH) in the oxidized MWCNT samples are responsible for the strong and wide peaks at 3433 cm^−1^ and 1725 cm^−1^. MWCNT backbone vibration is responsible for the peak at 1380 cm^−1^. The dispersion of MWCNT in liquids is enhanced by these oxygen-containing groups’ ion exchange capabilities [32]. New peaks at 1350 cm^−1^ corresponding to the C-N stretch are seen on the NH_2_-MWCNT and the HA-MWCNT spectra. Others are the –NH stretching vibration at 3450 cm^−1^ which overlaps with the –OH vibrations [33]. The FT-IR data show that the amine-functionalization and the hemin adsorption of the MWCNT surface were effective. 

The morphology of the MWCNT and the HA-MWCNT are presented in Figure 4c,d. The MWCNT were aggregated due to van der Waals forces. The length is in the range of a few microns and tens of microns, and the surface is reasonably smooth with an inner diameter ranging from 10 to 20 nm. After hemin adsorption, the HA-MWCNT still exhibited agglomeration with occasional granular clusters adhered to the surface, and the diameter increase somewhat, which may have been a result of the amine functionalization and the hemin adsorption.

The energy dispersive X-ray elemental distribution on the surface of MWCNT and the HA-MWCNT and the corresponding elemental mapping are depicted in Figure 4e–l. On the surface of MWCNT, a homogeneous distribution of atoms may be readily observed. Only carbon (C) and oxygen (O) are visible on the MWCNT, while nitrogen (N) and iron (Fe) are visible on the HA-MWCNT in addition to the C and N. Most of the carbon came from the carbon nanotubes, whereas the N and the Fe appeared due to the amine functionalization and the hemin adsorption, respectively.

**Figure 5:**
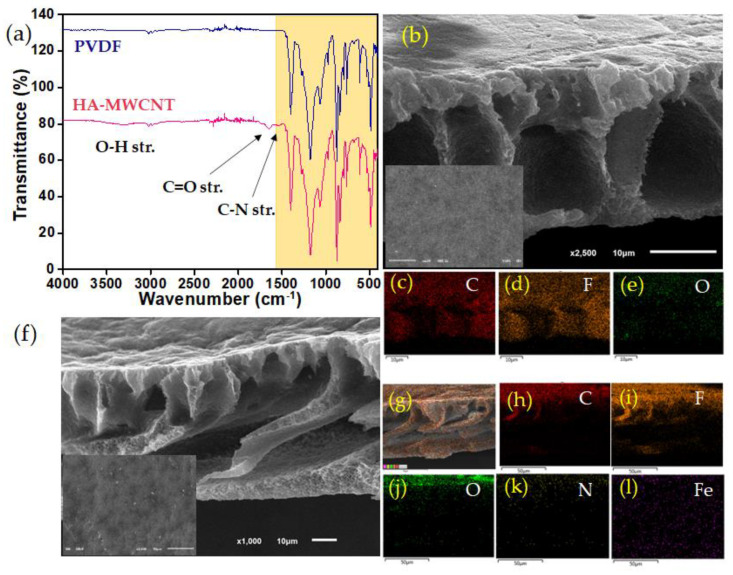
(**a**) The FTIR spectra of the PVDF and the HA-MWCNT membranes, (**b**) the FESEM of the PVDF membrane with the elemental mapping on the cross-section shown in (**c**–**e**). The morphology of the HA-MWCNT membrane is shown in (**f**), while the elemental mappings are shown in (**g**–**l**). The surface morphologies of both membranes are shown as insets on the cross-sectional views.

FTIR spectra of the PVDF and the HA-MWCNT membranes were investigated for the surface chemistry and to confirm the successful fabrication of the nanocomposite membrane. The results are displayed in Figure 5a. The –CH vibrations can be seen at 3022 and 2972 cm^−1^, while the –C–C– and the –C–F– vibrations appeared at 881 and 841 cm^−1^, respectively. The entire PVDF absorption bands in the region 500–1500 cm^−1^ can be seen on both spectra [34], except for the –C–N stretch, the –C=O stretch, and the -NH stretch which appeared at 1525, 1650, and 3450 cm^−1^, respectively, which appeared on the HA-MWCNT membrane, confirming the successful fabrication of the composite membrane.

The surface morphology and the cross-sectional view of the PVDF membrane is presented in Figure 5b, while the elemental mapping is shown in Figure 5c–e. Similarly, those of the HA-MWCNT membrane are depicted in Figure 5f–l. The surface morphology revealed that both membranes did not exhibit surface fracture during the synthesis, and that the addition of the MWCNT did not cause any agglomeration which limits the pore formation. Furthermore, the cross-sectional views revealed that both membranes exhibit an asymmetric finger-like framework with a dense top layer, typical of PVDF membranes [35]. The PVDF membrane, as predicted, has a smaller pore size compared to the HA-MWCNT membrane. Mixed-matrix membranes often have larger macro-void structures and higher overall porosity in the selective layer when compared to the pristine membranes [36]. In particular, the morphology was drastically altered when a specific amount of HA-MWCNT was added to the casting solution, resulting in macro-voids and pore diameter on the top that were much broader and larger than the pristine PVDF membrane**.** The elemental mapping thus reveals a uniform distribution of the atoms across the membranes.

**Figure 6:**
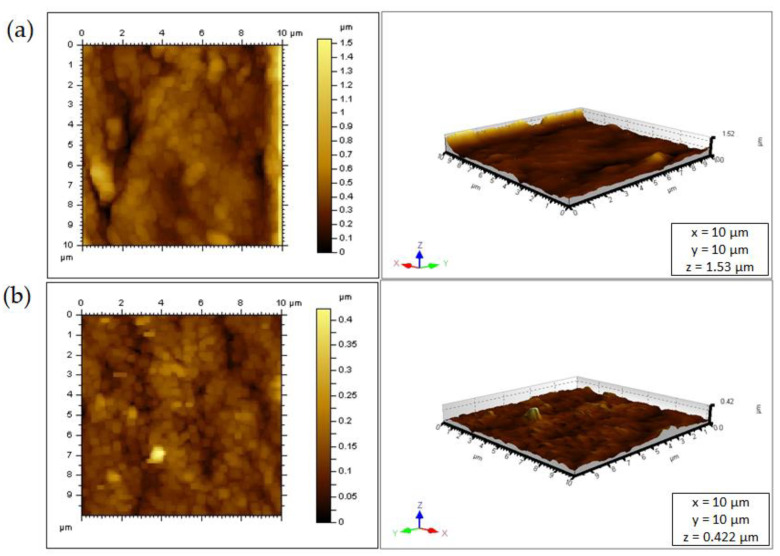
AFM micrographs of (**a**) PVDF and (**b**) HA-MWCNT membranes. The left and right images represent the 2D and the 3D topographies, respectively.

Surface roughness has an impact on a membrane’s antifouling performance. AFM analysis was used to measure the membranes’ surface roughness and the results are depicted in Figure 6a,b. The dark and bright spots on the 2D images represent pores and heights. An apparent difference in the topography and the surface roughness of the PVDF and the HA-MWCNT membranes was observed. The root-mean square roughness (*R*_q_) and the mean roughness (*R*_a_) were quite different, indicating the topography change after the addition of the HA-MWCNT. The pristine PVDF membrane formed by the phase inversion appeared much smoother. The mean surface roughness and root mean square roughness (*R*_q_) were estimated at 36.5 nm and 49.7 nm. These values correlate with those reported on PVDF membranes in the literature [37]. In the case of the HA-MWCNT membrane, the mean surface roughness and root mean square roughness were found to be 11.9 nm and 14.7 nm, respectively (Figure 6b). Thus, the modified membrane has a smoother surface. This can be attributed to the excellent dispersion and the low electrostatic interactions between the MWCNT particles [38]. 

**Figure 7:**
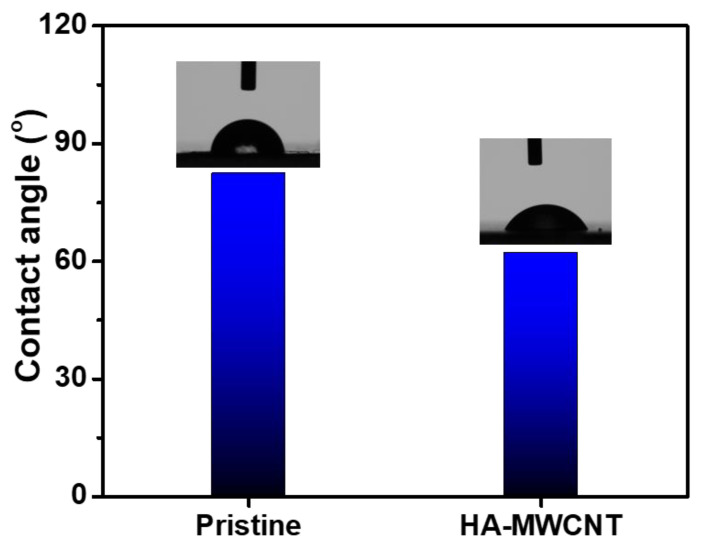
Water contact angle on the pristine and the HA-MWCNT membrane.

Meanwhile, the wettability of the membranes was measured and is depicted in Figure 7. For effective oil-in-water separations, the size exclusion principle plays a critical role as it provides an avenue to discriminate between oil and water molecules based on size [39]. However, to prevent the dispersed tiny oil droplets from penetrating and blocking the pores, the membrane’s hydrophilicity has to be controlled. The water contact angles on the PVDF and HA-MWCNT membrane surfaces were measured and the results revealed that the PVDF membrane has a value of 82°. The HA-MWCNT membrane on the other hand has a value of 62°. This considerable decrease can be attributed to the presence of polar –OH and –NH functional groups on the composite membrane which imparts surface hydrophilicity and lowers the fouling tendency.

**Figure 8:**
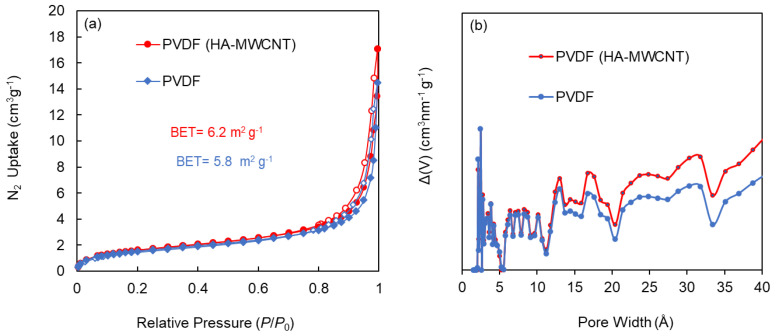
(**a**) N_2_ sorption isotherms at 77 K for HA-MWCNT (red) and pristine PVDF (blue). (**b**) Pore size distribution of HA-MWCNT (red) and pristine PVDF (blue).

Furthermore, the surface area and the pore diameter of the pristine and the HA-MWCNT membranes was evaluated with the help of the BET analysis. The N_2_ sorption isotherms at 77 K indicate that both membranes are mesoporous in nature (Figure 8a). The PVDF (HA-MWCNT) membrane showed enhancement in the pore diameter and higher uptake at high pressure (P/P0 ≥ 0.9); this also conformed to the pore size distribution (PSD) figure (Figure 8b).

**Figure 9:**
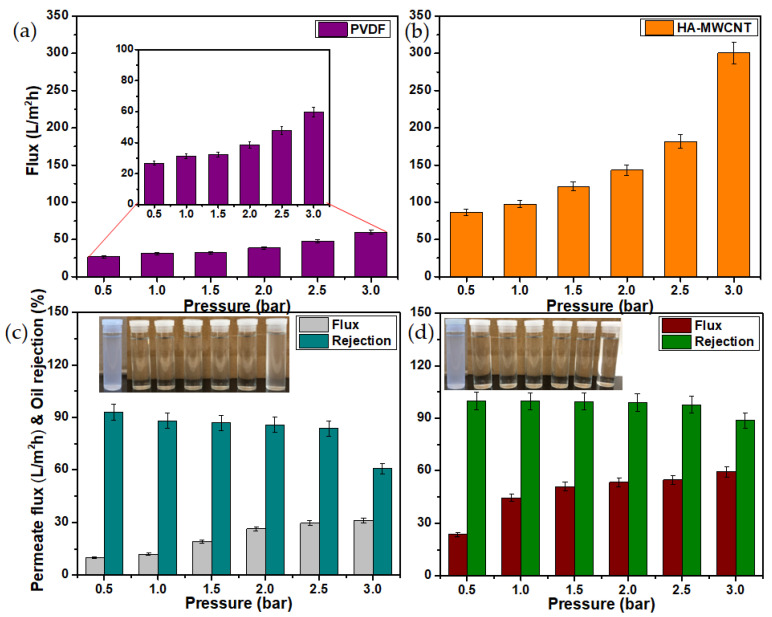
Water permeation flux on (**a**) pristine PVDF and (**b**) HA-MWCNT membranes. The permeate flux and the oil rejection on the pristine PVDF and the HA-MWCNT membranes are shown in (**c**,**d**), respectively. The snapshots of the emulsion feed and the permeate are shown as inset in (**c**,**d**).

The fabricated membranes were evaluated for the separation of oil-in-water emulsions and the results are presented in Figure 9. To assess their demulsification characteristics, the membranes (50 mm dia.) were fixed in a dead-end filtration cell and compacted for 1 h at 4.0 bar using deionized water. Thereafter, the flux of water through the membranes (*J_w_*) were evaluated at a transmembrane pressure range of 0.5–3.0 bar using the expression [40]:

$$J_{w}=\frac{V}{A\times \Delta t}\;$$

where *V*, *A*, and Δ*t* represents the permeation volume (m^3^), the membrane effective area (m^2^) and the permeation time (h), respectively. The pristine PVDF membrane recorded a steady increase in pure water flux reaching up to 59.9 L/m^2^h at 3.0 bar (Figure 9a). Similarly, the HA-MWCNT membrane showed an increase in water flux with an increase in transmembrane pressure (Figure 9b). However, the incorporation of the hemin-adsorbed amine-functionalized carbon nanotubes created additional pores and increased the hydrophilicity on the membrane surface which enhanced water permeation. Consequently, the HA-MWCNT membrane recorded a high pure water flux of 300.5 L/m^2^h at 3.0 bar. This represents a significant increment of about 401% compared to the pristine PVDF and showed a remarkable enhancement in water flux across the membrane.

Furthermore, the rejection of oil-in-water emulsions on the fabricated membranes was evaluated by passing a 200 ppm emulsion through the pristine PVDF and the HA-MWCNT membranes at pressures ranging from 0.5–3.0 bar. The emulsion was stabilized with the aid of sodium dodecyl sulfate (SDS) surfactant and the oil rejection (*R*%) across the membranes was estimated using the Equation [40]:

$$R\%=\left(\frac{C_{f}-C_{p}}{C_{f}}\right)\times 100$$

where *C_f_* and *C_p_* are the oil concentrations in the feed and in the permeate, respectively.

In contrast to the pristine PVDF membrane, the HA-MWCNT membrane showed excellent oil rejection of >99% in the transmembrane pressure range of 0.5–2.5 bar. Beyond this pressure, however, a slight decline in the oil rejection was observed and oil rejection decreased to 88.7% at 3.0 bar. It is worth mentioning that these results were achieved due to the unique design of the HA-MWCNT membrane which increased the hydrophilicity on the membrane surface and increases water accessibility while blocking the oil molecules, in good agreement with the MD simulation results. The performance of the PVDF membrane showed that the HA-MWCNTs incorporation into the PVDF membranes improved the flux and separation efficiency of the membrane. The HA-MWCNTs, provided the additional channel for permeation and improved the hydrophilicity of the PVDF membrane which result into the better flux and high rejection of the PVDF membrane.

**Figure 10:**
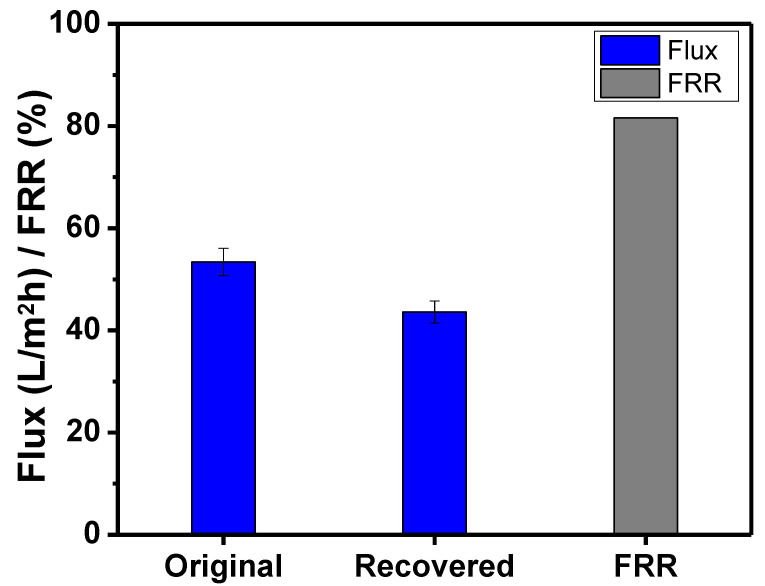
Flux recovery ratio on the HA-MWCNT membrane while separating 200 ppm of oil-in-water emulsions.

Membrane fouling during oil-in-water emulsion separation is a common phenomenon which often results in the blocking of the pores and a consequent decline in flux and oil rejection [35]. During the demulsification process, the large oil droplets aggregate at the membrane interface forming cake layers, while tiny droplets penetrate through the pores and block the channels. Both processes result in severe fouling of the membrane and lower its rejection efficiency. The fouling resistance of a given membrane is thus evaluated by analyzing its tendency to resist the cake formation and is expressed in terms of the flux recovery ratio (FRR) after exposure to the emulsion [35]. The resistance to fouling of the HA-MWCNT membrane was estimated by fixing the membrane in a dead-end cell and 100 mL of 200 ppm oil-in-water emulsion was added. A transmembrane pressure of 2.0 bar was applied to assess the antifouling behavior of the membranes and the membrane was kept exposed for the period of 3 h to the oil-in-water emulsions. Thereafter, the membrane was removed, thoroughly washed with deionized water, and mounted again in the cell and the pure water flux was measured. A steady decline in the permeation flux was observed during the 3 h operation. However, about 82% of flux was recovered after this process, indicating the fouling resistance of the HA-MWCNT membrane (Figure 10). When compared to other previously reported membrane materials, as listed in Table 1, the HA-MWCNT membrane achieved comparable results with excellent oil rejection of 99.1% at a transmembrane pressure of 2.0 bar, and an FRR of 82%.

## 3. Materials and Methods

### 3.1. Reagents and Chemicals

All the reagents and chemicals were of high purity and used as-received without further purifications. Sodium nitrite, NaNO_2_ (ACS reagent, ≥97.0%), sulfuric acid, H_2_SO_4_ (ACS reagent, 95.0–98.0%), chloro(protoporphyrinato)iron(III) (≥96.0%, HPLC), ethylene diamine (for synthesis), MWCNT (20–30 nm in diameter and 2–20 μm in length), N, N-dimethylformamide (DMF; 99.8% purity), N, N-dimethylacetamide (DMAc; ≥99.8%, GC), acetic acid (glacial, ACS reagent, ≥99.7%), sodium dodecyl sulfate (SDS; ACS reagent, ≥99.0%), ferric chloride hexahydrate (FeCl_3_·6H_2_O; ACS reagent, ≥97%), absolute ethanol, and methanol were procured from Sigma-Aldrich. The polyvinylidene fluoride, PVDF, was purchased from Alfa Aesar.

### 3.2. Synthesis of Chloro(protoporphyrinato)iron(III)-Adsorbed Amine-Functionalized MWCNT

#### 3.2.1. Synthesis of Amine-Functionalized MWCNT

The amine-functionalized MWCNT was synthesized following a previously reported method, with slight modifications [51]. Briefly, MWCNT (70 mg, 5.8 mmol of C) was mixed with NaNO_2_ (93 mg, 1.4 mmol) and ethylene diamine (85 mg, 1.4 mmol). Concentrated H_2_SO_4_ (0.061 mL, 1.2 mmol) was added and the mixture was heated at 60 °C for 1 h. The mixture was allowed to cool, then DMF was added and the mixture centrifuged and washed several times with DMF and water to remove any unreacted ethylene diamine from the product.

#### 3.2.2. Synthesis of Chloro(protoporphyrinato)iron (III)-Adsorbed MWCNT (HA-MWCNT)

**Scheme 1:**
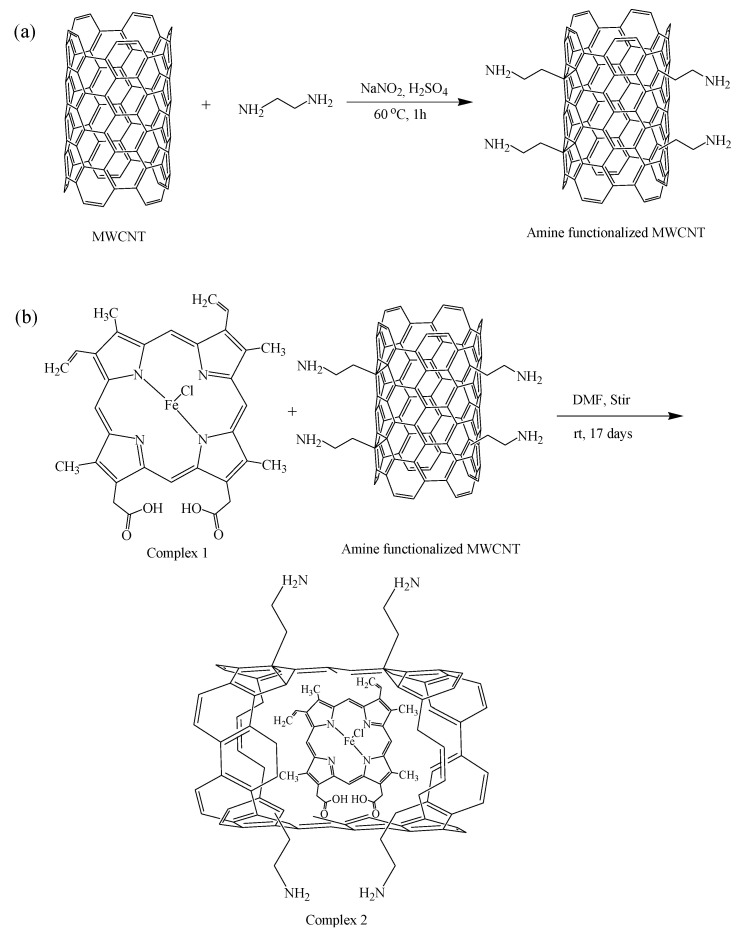
Synthesis route for (**a**) amine functionalization of MWCNT, (**b**) chloro(protoporphyrinato)iron (III) adsorbed onto amine-functionalized MWCNT.

Amine-functionalized MWCNT (10 mg, 0.83 mmol of C) was sonicated for 15 min in dry DMF (40 mL) to provide a dark suspension. Chloro(protoporphyrinato)iron (III) (complex 1) (5 mg, 0.005 mmol) was then added (resulting in a green colored suspension) and the mixture stirred for 17 days at which point the green color had faded (indicating the adsorption of complex 1 onto the MWCNT) [52]. The solid product was separated from the solution by centrifuging and washing several times with DMF to remove excess unreacted chloro(protoporphyrinato)iron (III) to obtain the adsorbed species: HA-MWCNT (complex 2) as a dark green powder. Scheme 1 presents an illustration of the synthesis.

### 3.3. Membrane Fabrication

The HA-MWCNT membrane was fabricated as follows [35]: the fine PVDF powder was kept at 60 °C under vacuum for 12 h to remove any adsorbed moisture. A calculated mass of the PVDF powder and HA-MWCNT was added to DMAc to obtain 18% casting solution of PVDF and 0.5% solution of HA-MWCNT. The solution was stirred at 60 °C overnight to obtain a homogeneous solution and later degassed for 1 h. After that, the obtained solution was kept for 24 h to remove any trapped bubbles. The PVDF solution was cast with the help of a doctor’s blade and immediately transferred into the coagulation bath. The coagulation bath consisted of deionized water. After 10 min, these membranes were moved into the deionized water and kept overnight for the complete phase inversion process. Afterward, the synthesized membranes were stored in deionized water for further evaluation. 

### 3.4. Characterizations

The morphologies of the multi-walled carbon nanotubes, the HA-MWCNT and the fabricated membranes were recorded on Quattro field emission scanning electron microscope (FESEM, Waltham, MA, USA) fitted with an energy dispersive X-ray spectrometer (Thermo Scientific^TM^, Waltham, MA, USA). The specimens were coated by gold sputtering prior to analysis. X-ray diffraction patterns were obtained on a Rigaku Miniflex-II X-ray diffractometer using CuKα radiation in the range of 2*θ* 5°–80°. The FTIR spectra of the materials and the fabricated membranes were measured on Nicolet^TM^ iS5 FTIR spectrometer in the range of wavenumber 400–4000 cm^−1^. The surface wettability of the fabricated membranes was measured on DSA-25 drop-shaped contact angle analyzer (KRUSS Scientific, Hamburg, Germany) using a drop size of 5 µL. The surface topography and roughness of the membrane specimens were recorded on Agilent 5500 atomic force microscope (Agilent Technologies, Santa Clara, CA, USA). Micrometric ASAP 2020 (Brussels, Belgium) was used to measure the BET surface area and pore size distribution. High-purity N_2_ (99.999) gas was used under the liquid nitrogen condition.

### 3.5. Oil–Water Separation

The fabricated membranes were evaluated for oil-in-water separation on a Sterlitech HP4750 high pressure dead-end filtration cell with an internal diameter of 5.1 cm and membrane active area of 14.6 cm^2^. Prior to the emulsion separation, the membranes were compacted at a specific pressure above the working pressure for 1 h. For the preparation of the oil-in-water emulsions, 1 L of the deionized water was taken and put on the magnetic stirrer at 700 rpm. The 1 g of SDS was added slowly to avoid the formation of foam in the deionized water and it was kept stirring for 15 min. After that, 1 g of oil was added and kept stirring until the milky color emulsion of 1000 ppm of oil-in-water emulsion formed. After that, it was diluted with the deionized water to obtain 200 ppm of emulsion. The emulsion droplet size was measured to be about 120 nm with the aid of the Nano-ZS ZEN 3600 Malvern zetasizer. The transmembrane pressures during the measurements were carefully controlled using a two-stage regulated argon gas flow. The demulsification efficiencies of the membranes were estimated using a turbidimeter which measures the turbidity of the feed and the permeate in NTU units [53,54].

### 3.6. Molecular Dynamics (MD) Simulations

**Figure 11:**
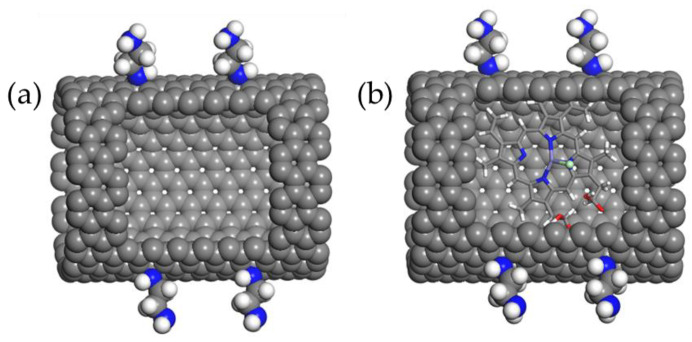
Models of (**a**) amine-functionalized MWCNT, (**b**) the HA-MWCNT membrane active layers.

Theoretical modeling of the demulsification of oil-in-water emulsions on the HA-MWCNT membrane was studied on Materials Studio 8.0 suite via the Forcite module. The universal forcefield which allows the prediction of geometries and conformational energy differences of organometallic systems was used [55]. The Connolly surface area and the fractional free volume (FFV) of the membrane systems were estimated by packing 7 units of the functionalized membrane materials (Figure 11a,b) in cubic amorphous cells with dimensions of 50 × 50 × 50 Å. The constructed cells were geometrically optimized, followed by dynamics simulations on the NPT and the NVT ensembles each for a duration of 1000 ps at a timestep of 10^−3^ ps. The Nose–Hoover thermostat and the Berendsen barostat were used to control the temperature and the pressure, respectively. Meanwhile, the Ewald summation method was used to treat the long-range Coulombic interactions, while the repulsive and attractive interactions were estimated using the Lennard–Jones potential at a cutoff range of 18.5 Å.

For the stability at the oil–water interface in the system containing the membrane material, *HA-MWCNT* was estimated by the construction of symmetric cells comprising 1000 water molecules, 5 units of *HA-MWCNT*, 5 molecules of sodium dodecyl sulfate (*SDS*) which act as the emulsifier, and 200 molecules of octane which represent the oil model. Similar cells without the emulsifier were also constructed. All the cells were geometrically optimized, and thereafter subjected to NPT and NVT dynamic simulations with periodic boundary conditions each for 500 ps at 298.5 K. All other simulation conditions remained the same as previously mentioned. The oil–water interface stability was estimated by calculation of the interfacial formation energy (*E_IFE_*) using the equation [56]:

$$E_{IFE}=\frac{1}{2}\left[E_{system}-\left(E_{HA-MWCNT}+E_{SDS}+E_{oil-water}\right)\right]$$

where $E_{system}$, $E_{HA-MWCNT}$, $E_{SDS}$ and $E_{oil-water}$ (kcal/mol) represents the total energies of the configuration under study, the *HA-MWCNT* membrane active layer, the *SDS* emulsifier, and the *oil–water* system, respectively.

Lastly, we conducted MD simulations to assess the interactions between the hydrocarbon units, water molecules, and the membrane active layer. Amorphous simulation cells with dimensions of 40 × 40 × 40 Å were constructed comprising a unit of the membrane active layer, 5 molecules each of hexane, octane, and dodecane, and 1000 molecules of water. The periodic boundary conditions were set in all directions and the hydrocarbons each was positioned at distances at least 5.0 Å from the membrane surface. Following the energy minimizations, and the NPT and NVT dynamics simulations for 500 ps (conditions remain the same as previously mentioned), the interactions of the hydrocarbon molecules with the HA-MWCNT membrane active layer were estimated.

## 4. Conclusions

We report the successful fabrication of a hemin-modified MWCNT-incorporated PVDF membrane (HA-MWCNT) to separate oil-in-water emulsions. The fabricated membrane exhibited an asymmetric finger-like morphology, solvent-accessible macro voids, and surface roughness of 11.9 nm compared to the pristine PVDF, which recorded a roughness of 36.5 nm. In contrast to the PVDF membrane, the HA-MWCNT achieved a water contact angle of 62° ± 0.5, excellent pure water permeance of 300.5 L/m^2^h at 3.0 bar and maintained oil rejection of >99% in the transmembrane pressure range of 0.5–2.5 bar. The flux recovery of the hemin-modified MWCNT-incorporated PVDF membrane was greater than 80%. These results were consistent with those of computational modeling using classical MD simulations. Our results reveal that the incorporation of hemin-modified MWCNT during the fabrication of PVDF membranes is an excellent strategy to improve the fouling resistance and water permeance and could be adopted in designing advanced membranes for oil/water separation

## Figures and Tables

**Table 1:** Performance comparison of the HA-MWCNT membrane with previously reported membranes.

Membrane	Fabrication Method	Rejection (%)	FRR (%)	Ref.
PVFD + dopamine	Inkjet printing	99	82	[37]
PVDF + Cellulose	Supramolecular adhesive	99	80	[41]
PVDF + grafted poly(N-acryloylmorpholine	Surface-initiated atom transfer radical polymerization	91	50	[42]
polyacrylonitrile (PAN) membrane	Phase inversion	98.7	9.6	[43]
PSf/HAO	Phase inversion	100	67	[44]
Hydrophilic Fe_2_O_3_ dynamic membrane	Pre-coated dynamic membrane	95	53.7	[45]
Nanocomposite Kevlar fabric membranes	Dip coating	89.06	88.88	[46]
Isotropic polyether sulfone (PES) membrane	Non-solvent vapor-induced phase separation (VIPS)	98	59.5	[47]
PES/PDA@ZnFe_2_O_4_	Non-solvent induced phase separation (NIPS) process	96	82.5	[48]
PES/γ-Fe_2_O_3_	Phase inversion	82	56	[49]
PPSU/PEI	Phase inversion	56	42.6	[50]
HA-MWCNT	Phase inversion	99.1	81.6	Present study

## Data Availability

The data presented in this study are available on request.

## Sample Availability

Samples of the compounds are not available from the authors.
